# Patient-derived primary breast cancer cells and their potential for predicting sensitivity to chemotherapy

**DOI:** 10.3389/fonc.2022.1023391

**Published:** 2022-10-14

**Authors:** Yajun Mou, Jianjun Huang, Wenxiu Yang, Yu Wan, Zhenhong Pu, Junhong Zhang, Jinting Liu, Qing Li, Peipei Zhang, Yuan Tian, Hui Yang, Yi Cui, Pingsheng Hu, Xiaowei Dou

**Affiliations:** ^1^ Department of Pathology, The Affiliated Hospital of Guizhou Medical University, Guiyang, China; ^2^ Clinical Research Center, The Affiliated Hospital of Guizhou Medical University, Guiyang, China; ^3^ Department of Breast Surgery, The Affiliated Hospital of Guizhou Medical University, Guiyang, China; ^4^ Department of Orthopaedics, The Affiliated Hospital of Guizhou Medical University, Guiyang, China

**Keywords:** primary cells, breast cancer, pirarubicin, doxorubicin, sensitivity

## Abstract

Chemotherapy resistance exposes patients to side effects and delays the effect of therapy in patients. So far, there are no predictive tools to predict resistance to chemotherapy and select sensitive chemotherapeutic drugs for the patient. Here, we aim to develop an *in-vitro* primary cell culture model from breast cancer patients to predict sensitivity to chemotherapy. We created the primary breast cancer cell medium BCMI and culture system with higher efficiency of the model establishment. Immunofluorescence staining of ERa, PR and HER2 were done to identify the primary breast cancer cell from the counterpart breast cancer patient. The killing assay showed that these primary breast cancer cells responded differently to doxorubicin and pirarubicin treatment. These results indicate that our established primary breast cancer cell model holds great promise for predicting breast cancer sensitivity to chemotherapy drugs.

## Introduction

Among women, breast cancer is the most commonly diagnosed cancer and the leading cause of cancer mortality worldwide in 2020, with an estimated 2.3 million newly diagnosed cases and 685,000 deaths (1). Neoadjuvant chemotherapy (NAT) is increasingly being utilized as the first-line therapy for high-risk, locally advanced, or unresectable breast cancers (2). However, the response to NAT differs by breast cancer subtypes. The Collaborative Trials in Neoadjuvant Breast Cancer (CTNeoBC) group analyzed 12 pooled trials and concluded that the pathologic complete response (pCR) rates after NAC were also high in TNBC (33.6%), HER2-positive breast cancer (30.2%), grade 1/2 HR-positive breast cancer (7.5%), and grade 3 HR-positive breast cancer (16.2%) (3). In the I-SPY trial, the rates of pCR were the lowest for HR-positive, ERBB2-negative tumors (17.4%) and up to 68% for HR-negative, ERBB2-positive tumors. The trial also found that achieving pCR after neoadjuvant therapy implies approximately an 80% reduction in the recurrence rate of breast cancer regardless of the subtype and/or treatment regimen (4). Therefore, it is crucial to rapidly and predictively identify drugs with the greatest activity from various regimens of standard chemotherapy for improving breast cancer pCR rate, particularly for HR-positive breast cancer.

So far, there is no specific tool to predict breast cancer sensitivity in clinic. Through investigation, gene expression profiles were found to be correlated with neoadjuvant chemotherapy efficacy, but more data are needed to confirm the correlation between gene expression profiles and chemotherapy efficacy (5–8). Patient-derived primary cancer cells can be used to identify effective drug combinations for cancer, but the success rate was only 15% for generating primary breast cancer cells (9, 10). Cancer organoid culture is developed to assess breast cancer sensitivity to drugs for individual patients, but more research should address the optimization of the organoid culture and the concordance between drug responses and actual clinical outcomes (11–13). Patient-derived xenograft models (PDX) of breast cancer have been generated to recapitulate the diversity of breast cancer and predict drug response. However, current PDX models cannot accurately predict drug efficacy because of the different tumor microenvironments from the original tumor (14–16). Hence, models for predicting personalized breast cancer sensitivity to chemotherapy need to be created.

Here, we created a primary breast cancer cell model for predicting individual breast cancer sensitivity to chemotherapy. We were successful in creating the final breast cancer cell lines in 77% of breast cancer samples. The killing assay showed that these primary breast cancer cells responded differently to pirarubicin and doxorubicin treatment. Thus, our established primary breast cancer cell model has the potential to predict breast cancer sensitivity to chemotherapy clinically.

## Results

### Culture of primary cancer cells from breast cancer tissues

**Figure 1:**
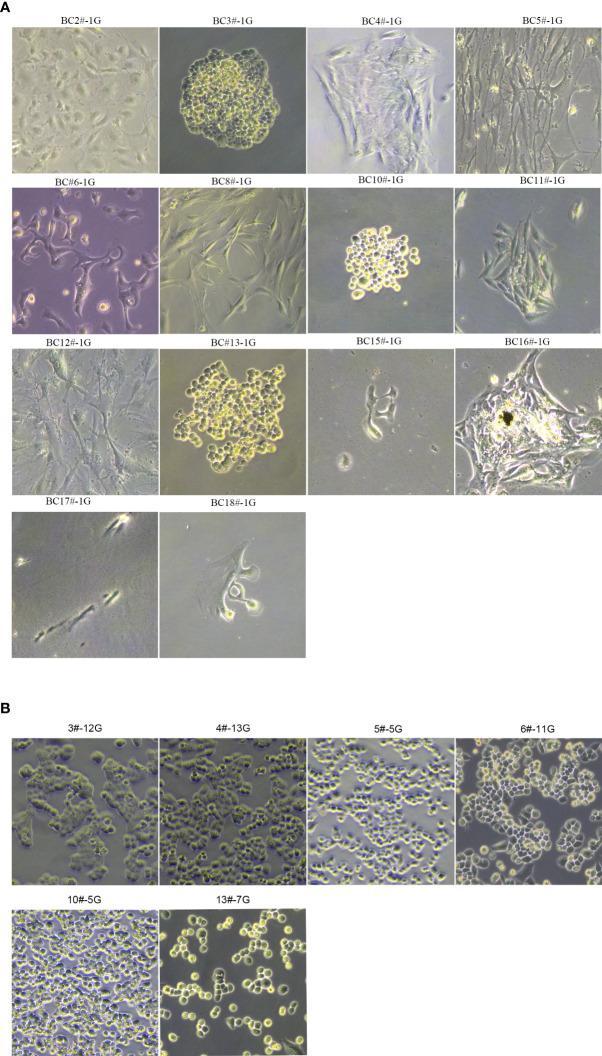
The morphology of primary breast cancer cells. **(A)** BC2#-1G, BC3#-1G, BC4#-1G, BC5#-1G, BC6#-1G, BC8#-1G, BC10#-1G, BC11#-1G, BC12#-1G, BC13#-1G, BC15#-1G, BC16#-1G, BC17#-1G and BC18#-1G. **(B)** The morphology of primary breast cancer cells: BC3#-12G, BC4#-13G, BC5#-5G, BC6#-11G, BC10#-5G, and BC13#-7G (magnification Number ×100). # means patient. G means generation.

Based on our previously established breast stem cell medium MaECM, we created the breast cancer medium BCMI (17). Since December 2018, we have attempted to generate primary cancer cells from breast cancer patients’ biopsies or surgical tissues. The samples were obtained from the biopsies or surgical tissues after neoadjuvant treatment and minced using blades. After being digested with 280 U/ml of collagenase II at 37°C for 1 h, the cells were filtered using a 70-μM strainer. The primary breast cancer cells were cultured in the medium BCMI. When the cells proliferated speedily, the medium DMEM was used to culture the primary breast cancer cells. A finished culture is one in which the cancer cells passaged infinitely and can be cryopreserved, thawed, and regrown. Using these criteria, 14 breast cancer cell lines were generated from 18 breast cancer patient tissues, with a success rate reaching 77% (see **Figure 1**; **Table 1** ,and **Figure S1** ).

**Table 1:** Success of primary breast cancer cells generation.

Tumor type	Number finished	Number failed	Total processed	Percent successful
Breast cancer	14	4	18	77%

### Verification of the origination of the primary breast cancer cells

**Figure 2:**
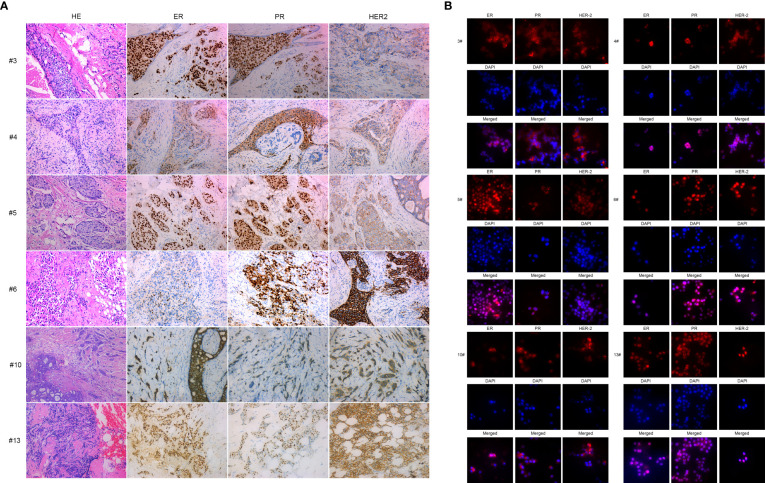
IHC and immunofluorescence staining of primary breast cancer cells: BC3, BC4, BC5, BC6, BC10, and BC13. **(A)** IHC staining of ER, PR, and HER2 of BC3, BC4, BC5, BC6, BC10, and BC13 breast cancer tissue sections. **(B)** Immunofluorescence staining of ER, PR, and HER2 in the primary breast cancer cells BC3, BC4, BC5, BC6, BC10, and BC13.

To confirm primary breast cancer cells from breast cancer tissues, we stained ERα, PR, and HER2 in six kinds of primary breast cancer cells and their correlated breast cancer tissues. Interestingly, the expression of ERα, PR, and HER2 in primary breast cancer cells was concordant with the expression of molecular markers in the counterpart breast cancer tissues (see **Figures 2A, B** and **Table 2**).

**Table 2:** The expression of molecular markers in 6 Cases of Patients with Breast Cancer.

			
markers	ER	PR	HER2
cases			
3#	90%	70%	1+
4#	50%	60%	1+
5#	80%	70%	2+
6#	20%	40%	2+
10#	80%	70%	1+
13#	80%	50%	2+

The symbol "+" means Her2 expression level.

### A model to predict breast cancer sensitivity to pirarubicin

**Figure 3:**
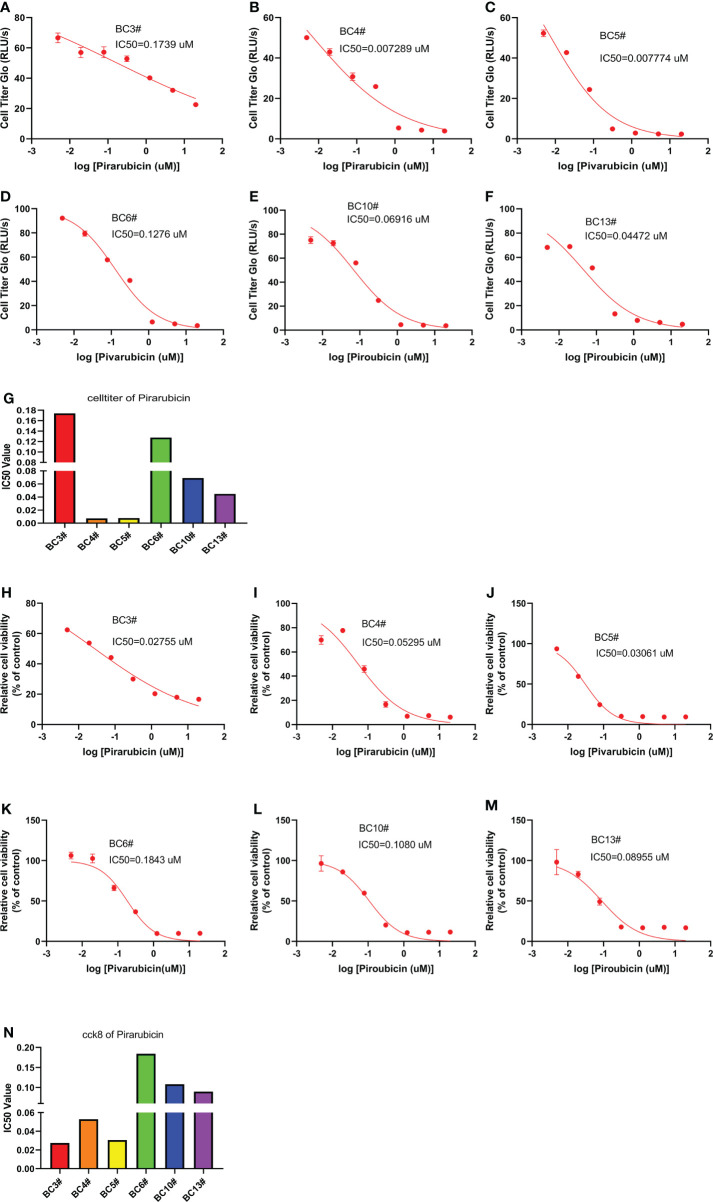
The killing assay of pirarubicin by the CellTiter-Glo and CCK8 assays. **(A–G)** The CellTiter-Glo assay to measure the IC_50_ value of pirarubicin in the primary breast cancer cells BC3, BC4, BC5, BC6, BC10, and BC13. **(H–N)** The CCK8 assay to measure the IC_50_ value of pirarubicin in the primary breast cancer cells BC3, BC4, BC5, BC6, BC10, and BC13.

In order to predict the sensitivity of breast cancer cells to pirarubicin, six primary breast cancer cells were used to measure the sensitivity of breast cancer cells to pirarubicin using the CellTiter-Glo and CCK8 assays. Intriguingly, the CellTiter-Glo assay results showed that these primary breast cancer cells responded differently to pirarubicin. BC3# and BC6# cells have higher IC_50_ values of pirarubicin, but BC4# and BC5# cells have lower IC_50_ values of pirarubicin, indicating that breast cancer cells BC4# and BC5# are sensitive to pirarubicin treatment (**Figures 3A–G**). Similarly, the CCK8 assay results showed that BC6# cell has a higher IC_50_ value of pirarubicin, but BC4# and BC5# cells have lower IC_50_ values of pirarubicin (**Figures 3H–N**). However, we noted that BC3# cell has a higher IC_50_ value of pirarubicin in the CellTiter-Glo assay but a lower IC_50_ value of pirarubicin in the CCK8 assay. Since the CellTiter-Glo assay is used to determine the number of viable cells based on quantitation of the ATP present which is directly proportional to the number of cells and the CCK-8 assay is used to detect the dehydrogenase activity in viable cells and may cause a discrepancy between the actual viable cell number and the cell number determined, we think that breast cancer BC3# cell is resistant to pirarubicin treatment. Therefore, primary breast cancer cells can be used to detect breast cancer sensitivity to pirarubicin treatment.

### A model to predict breast cancer sensitivity to doxorubicin

**Figure 4:**
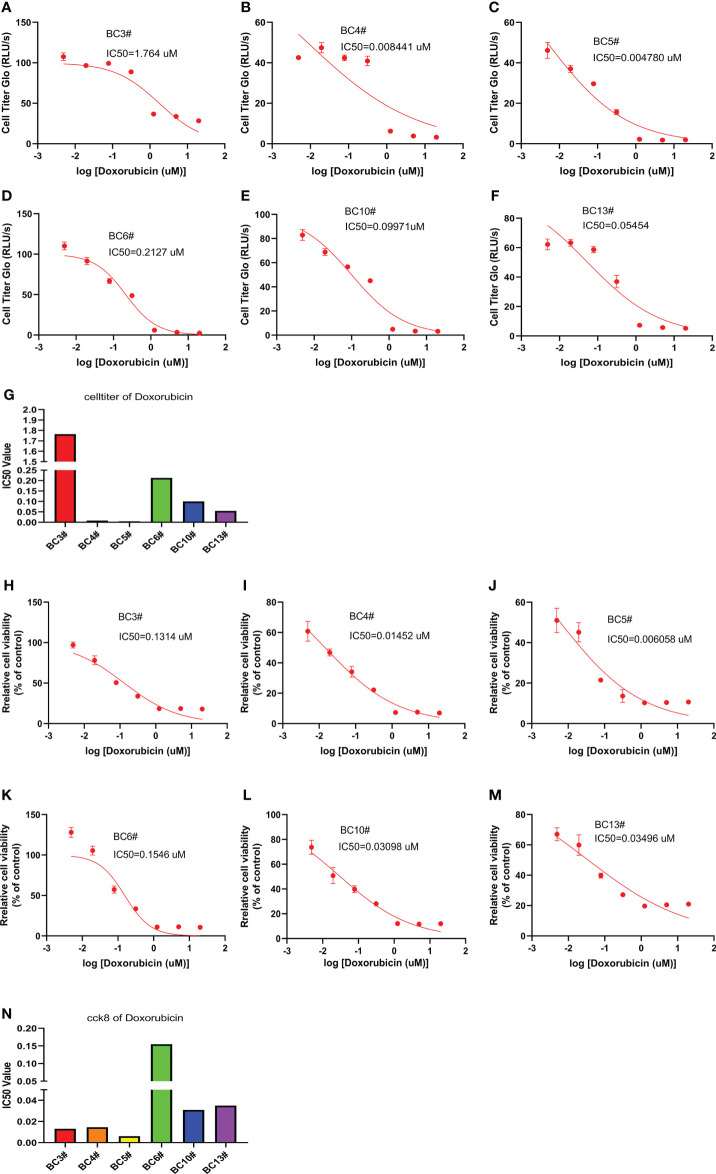
The killing assay of doxorubicin by the CellTiter-Glo and CCK8 assays. **(A–G)** The CellTiter-Glo assay to measure the IC_50_ value of doxorubicin in the primary breast cancer cells BC3, BC4, BC5, BC6, BC10, and BC13. **(H–N)** The CCK8 assay to measure the IC_50_ value of doxorubicin in the primary breast cancer cells BC3, BC4, BC5, BC6, BC10, and BC13.

We also predict the sensitivity of breast cancer cells to doxorubicin in six primary breast cancer cells using the CellTiter-Glo and CCK8 assays. The CellTiter-Glo assay confirmed the different responses of primary breast cancer cells to doxorubicin treatment. The IC_50_ values of doxorubicin were higher in BC3# and BC6# cells but lower IC_50_ values of doxorubicin in BC4# and BC5# cells, indicating that breast cancer BC3# and BC6# cells exhibited low sensitivity to doxorubicin treatment (**Figures 4A–G**). Similarly, the CCK8 assay results confirmed that BC6# cell has a higher IC_50_ value of doxorubicin, but BC4# and BC5# cells have lower IC_50_ values of doxorubicin (**Figures 4H–N**). Similarly, we noted that BC3# cell exhibited a higher IC_50_ value of doxorubicin in the CellTiter-Glo assay but a lower IC_50_ value of doxorubicin in the CCK8 assay. Based on a previous analysis, we think that breast cancer BC3# cell is resistant to doxorubicin treatment. Thus, primary breast cancer cells can be used as a tool to detect the sensitivity of breast cancer cells to doxorubicin treatment.

## Discussion

In the present study, we developed a primary breast cancer cell culture system and achieved a 77% success rate. We identified the source of primary breast cancer cells from breast cancer patient tissues using similar expressions of the molecular markers ER, PR, and HER2. We found that these primary breast cancer cells respond differently to pirarubicin or doxorubicin treatment. Basically, we created a primary breast cancer cell model which can be used to detect chemotherapy sensitivity *in vitro*.

The success rate of primary breast cancer cell culture is still the major obstacle for utilizing it to predict breast cancer sensitivity to chemotherapy. Previous research showed that, from 104 breast cancer patient tissues, 16 breast cancer cells were cultured successfully and 88 breast cancer tissues failed to culture cells, with a success rate only reaching 15% (10). Based on the culture of mouse mammary stem cells, we created a 77% success rate of primary breast cancer cell culture in our research (17). The higher success rate of the primary breast cancer cell culture renders the possibility of utilizing these primary breast cancer cells to predict chemotherapy sensitivity *in vitro*.

The culture condition for primary breast cancer cell culture is another major obstacle for utilizing it to predict breast cancer sensitivity to chemotherapy. The investigators found that the irradiated fibroblast feeder cells + TCM (tumor culture media) condition remains to be the optimized media in generating a cancer cell line (10). The irradiated fibroblasts and their produced growth factors have been identified to mediate resistance to targeted therapies (18–20). Exogenous EGF and insulin in TCM media could reduce the sensitivity of the EGFR mutant and ALK-translocated NSCLC cells to EGFR or ALK inhibitors, respectively (10). In our culture system, there were no irradiated fibroblast feeder cells and the exogenous growth factors EGF/insulin in the DMEM medium. Our results revealed that the primary breast cancer cells respond differently to pirarubicin or doxorubicin treatment. Therefore, our established primary breast cancer system may be used to predict breast cancer sensitivity to chemotherapy *in vitro*.

In conclusion, we were able to create breast cancer cells from breast cancer patient tissues, with a success rate reaching 77%. Using the primary breast cancer cell model, we found that these primary breast cancer cells responded differently to pirarubicin and doxorubicin treatment. Although we established the breast cancer cell culture system and use it for measuring chemodrug efficacy, there are still some issues to be solved. First, the primary breast cancer cells and the counterpart patient tissues should be sequenced for the confirmation of the cell source. Second, more primary breast cancer cells should be established to test the concordance between drug responses and actual clinical outcomes. Third, the definite concentration of the chemodrug should be determined in the breast cancer cell model to decide whether or not the patient is sensitive and can be treated by the chemotherapeutic drugs.

## Method and materials

### Tumor sample processing and cell culture

All human breast cancer samples were obtained from patients, with their informed consent, from the Affiliated Hospital of Guizhou Medical University, and all procedures were approved by the Ethics Committee of the Affiliated Hospital of Guizhou Medical University. The patients’ clinicopathological status is listed in **Table 3**. The biopsies and resections from patients were placed in sterile 15-ml centrifuge tubes containing D-Hank’s Balanced Salt Solution with 500 U/ml of penicillin and 500 μg/ml of streptomycin when transporting from the operating room to the research laboratory. The tumor tissues were placed at 4°C before processing within 24 h after resection. Biopsies and resections were minced with a sterile scalpel and then transferred into enzymatic digestion with 140 U/ml of collagenase II (17101015, Thermo Fisher Scientific, USA) in D-Hank’s Balanced Salt Solution in a 37°C shaker set at 100 rpm for 1 h. After digestion, the cells were centrifuged at 1,000 rpm for 10 min. The cells were filtered through a 70-μm strainer and cultured in BCMI media in a 5% CO_2_ incubator at 37°C. After being passaged three times, the medium was changed to DMEM media containing 10% FCS (Gibco), 100 U/ml of penicillin, and 100 μg/ml of streptomycin.

**Table 3:** Clinicopathological status in 6 cases of patients with breast cancer.

Cases	3#	4#	5#	6#	10#	13#
Variables						
**Age**	68	45	44	47	46	37
**Menopausal status**	Post-	Post-	Pre-	Pre-	Pre-	Pre-
**Size of tumor(mm)**	28*6	40*33*12	15*14*19	36*15*28	50*29*39	26*14*26
**TNM**	T2N2M0	T2N0M0	T2N0M0	T4N0M0	T4N1M0	T2N1M0
**Pathological grade**	III	I	II		II	II
**Lymph node status**	Positive	Positive	Negative	Positive	Positive	Negative
**Metastasis**	Negative	Negative	Negative	Negative	Negative	Negative

Number# Indicates the patient. *Means multiply.

### IHC staining

Immunohistochemistry (IHC) staining was done as previously described (21). Briefly, the sample was processed by deparaffinization, rehydration, antigen retrieval, and IHC staining. The primary antibodies used in immunohistochemistry staining were all purchased from Zhongshan Golden Bridge Biotechnology Company Ltd. (China).

### Immunofluorescence staining

Immunofluorescence staining was performed as previously done (17). Briefly, 5 * 10^4^ cells were seeded in each well of Millicell EZ SLIDE eight-well glass (Millipore), fixed with 4% PFA, and permeabilized with 0.1% Triton X-100. After blocking with 2.5% goat serum, the cells were incubated with rabbit anti-human estrogen receptor α antibody (D8H8, 1:1,600, CST), rabbit anti-human progesterone receptor A/B (D8Q2J, 1:800, CST), or rabbit anti-human HER2/ErbB2 (D8Q2J, 1:400, CST) overnight at 4°C. After rinsing with phosphate-buffered saline (PBS), anti-rabbit IgG (Alexa Fluor 555 conjugate, CST) was used to incubate the cells for 30 min. Alexa Fluor 555-conjugated rabbit IgG (Alexa Fluor 555 conjugate, CST) was used as the negative control. The cells were then rinsed with PBS three times and stained with DAPI (CST) for 1 min. A Zeiss Axiocam fluorescence microscope was used for image acquisition and analysis.

### CCK8 assay

BC cells (1 * 10^4^) were plated in each well of a 96-well plate. Each was performed in triplicate. After 1 day of culture, the cells were cultured in a 200 ul medium with 20, 5, 1.25, 0.312, 0.078, 0.0195, 0.0488, 0.00122 and 0 uM Doxorubicin or Pirarubicin. The cytotoxicity assay was performed using the Cell Count Kit-8 (Dojindo, Japan). After 72 h of culture, the cells were incubated in an incubator with CCK8 for 1 h at 37°C and the absorbance was measured at 450 nm using a microplate reader (SynergyH4, BioTek).

### CellTiter-Glo assay

The processing of cells was similar to the CCK8 assay. The viability assay was detected using the CellTiter-Glo Luminescent kit (Promega, USA). The experiment was performed according to the manufacturer’s instructions. After 72 h of culture with doxorubicin, pirarubicin, or docetaxel, 100 μl of CellTiter-Glo reagent was added into a 100-μl medium of each well of a 96-well plate, mixed well on an orbital shaker for 2 min, and incubated at room temperature for 10 min. Luminescence was measured by a microplate reader (SynergyH4, BioTek).

## Data availability statement

The original contributions presented in the study are included in the article/supplementary material. Further inquiries can be directed to the corresponding author.

## Ethics statement

The studies involving human participants were reviewed and approved by Ethics Committee of the Affilated Hospital of Guizhou Medical University. The patients/participants provided their written informed consent to participate in this study.

## Author contributions

YM, PZ, YT, HY, and YC contributed to the cell culture and assays. WY, ZP, and JZ contributed to the pathological analysis. JH, YW, JL, and QL and PH contributed to the clinical analysis. JH and XD devised the research. XD wrote the manuscript. All authors contributed to the article and approved the submitted version.

## Funding

This research was funded by the National Natural Science Foundation of China (No. 81860516).

## Conflict of interest

The authors declare that the research was conducted in the absence of any commercial or financial relationships that could be construed as a potential conflict of interest.

## Publisher’s note

All claims expressed in this article are solely those of the authors and do not necessarily represent those of their affiliated organizations, or those of the publisher, the editors and the reviewers. Any product that may be evaluated in this article, or claim that may be made by its manufacturer, is not guaranteed or endorsed by the publisher.
